# Evolution of functional antibodies following acute Epstein-Barr virus infection

**DOI:** 10.1371/journal.ppat.1010738

**Published:** 2022-09-06

**Authors:** Christina B. Karsten, Yannic C. Bartsch, Sally A. Shin, Matthew D. Slein, Howard M. Heller, Kumaran Kolandaivelu, Jaap M. Middeldorp, Galit Alter, Boris Julg

**Affiliations:** 1 University of Duisburg-Essen, University Hospital Essen, Institute for Translational HIV Research; Essen, Germany; 2 Ragon Institute of MGH, MIT and Harvard; Cambridge, Massachusetts, United States of America; 3 MIT; Cambridge, Massachusetts, United States of America; 4 MIT Institute for Medical Engineering & Science; Cambridge, Massachusetts, United States of America; 5 VU University Medical Center; Amsterdam, Netherlands; University of Zurich, SWITZERLAND

## Abstract

While Epstein-Barr virus causes mostly asymptomatic infection, associated malignancies, and autoimmune and lymphoproliferative diseases occur. To dissect the evolution of humoral immune responses over the course of EBV infection and to gain a better understanding of the potential contribution of antibody (Ab) function to viral control, we comprehensively profiled Ab specificities and Fc-functionalities using systems serology and VirScan. Ab functions against latent (EBNA1), early (p47/54) and two late (gp350/220 and VCA-p18) EBV proteins were overall modest and/or short-lived, differing from humoral responses induced during acute infection by other viruses such as HIV. In the first year post infection, only p18 elicited robust IgM-driven complement deposition and IgG-driven neutrophil phagocytosis while responses against EBNA-1 were largely Fc-functionally silent and only matured during chronic infection to drive phagocytosis. In contrast, Abs against Influenza virus readily mediated broad Fc-activity in all participants. These data suggest that EBV evades the induction of robust Fc-functional Abs, potentially due to the virus’ life cycle, switching from lytic to latent stages during infection.

## Author summary

While previously thought to be largely innocuous, emerging data clearly highlight the pathological role of lifelong EBV infection in driving autoimmunity and malignancies in a small, but not insignificant portion of the population. We therefore aimed to define potential humoral mechanisms associated with viral control, beyond neutralizing Abs, by systematically focusing on antibody Fc-functional activities during acute to convalescent EBV infection applying technologies such as systems serology and VirScan. We found that functions against EBV proteins were overall only modest and either short-lived or delayed, differing from functional antibody responses induced during acute infection by other viruses such as HIV. These data suggest that EBV evades the induction of robust Fc-functional Abs thereby potentially facilitating lifelong, persistent infection with all its consequences.

## Introduction

Epstein-Barr virus (EBV), like other herpesviruses, causes latent infection and it is estimated that more than 90% of the world’s population is EBV-seropositive [1]. Primary infection occurs in the majority of individuals in the first two decades of life, can be asymptomatic but can also cause infectious mononucleosis (IM). While IM is usually a self-limiting disease with characteristic symptoms of fever, pharyngitis and lymphadenopathy, it is medically significant due to the duration of the disease of several weeks and the number of affected individuals [2]. In addition, EBV infection has also been linked to a wide range of malignancies, including Hodgkin’s lymphoma (HL), Non-Hodgkin’s lymphoma, specifically Burkitt’s lymphoma, nasopharyngeal carcinoma (NPC), and gastric carcinoma but also posttransplant lymphoproliferative diseases (PTLDs) in immunocompromised hosts (reviewed in [3]) and more recently to multiple sclerosis [4,5]. Specifically, with the increasing usage of immune-modifying therapies for the treatment for autoimmune disorders and the increasing numbers of organ transplantations requiring immunosuppressive regimens, EBV-infection remains a complicating factor.

While acute IM induces a robust T-lymphocyte response [6], and binding antibodies (Abs) to viral capsid antigen and early antigen complex are readily found [7], little to no EBV-specific neutralizing Abs are detected during primary infection [8]. In fact, neutralizing Abs, i.e. against the glycoprotein gp350, only appear once individuals enter the convalescent phase of the infection. In addition to neutralization, however, Abs can mediate a multitude of functions via their fragment crystallizable region (Fc region), including recruitment and activation of innate effector cells via the engagement of Fc gamma receptors (FcgR) on the surface of these cells. Functions like Ab-dependent cell-mediated cytotoxicity (ADCC), Ab-dependent cellular phagocytosis by monocytes (ADCP) or neutrophils (ADNP), complement-dependent cytotoxicity (CDC), and anti-inflammatory activities have been proposed to play a role in protection against and control of multiple infections including HIV, Influenza-virus, Ebola virus etc. (reviewed in [9]). Pioneering work has demonstrated NK cell–mediated ADCC against EBV-infected cells [10–12] and a recent study confirmed that Abs from EBV-seropositive individuals can trigger NK cell degranulation and cytokine production [13]. Additionally, antibody responses mediating ADCP, were found during chronic but not acute EBV infection [14]. Furthermore, a recent study in a cohort of Ugandan infants reported that maternal neutralizing Abs did not protect against EBV-infection and a potential protective role of non-neutralizing Ab (nnAb) functions was suggested [15]. Given the accumulating hypotheses, pointing to a potential role of nnAb functions during EBV-infection, we conducted a comprehensive Ab profiling approach, using systems serology [16] and VirScan [17] to dissect the landscape of the EBV humoral immune response in a cohort of students, that had been followed from the acute to the convalescent stage of EBV infection.

## Results

### Clinical characteristics of study cohort

**Fig 1:**
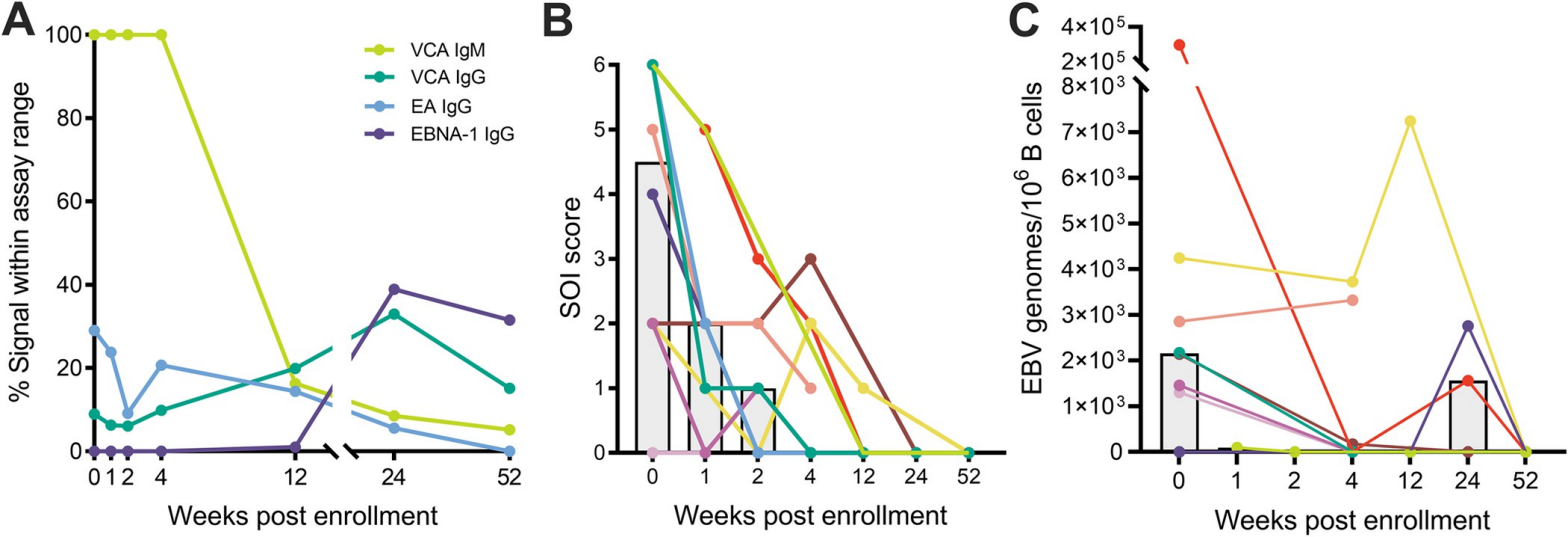
Clinical cohort characteristics. **(A)** Cohort participant’s antibody responses to EBV were measured by standard diagnostic ELISA over the sampling period of one year. Assay signals were normalized to the measurable signal range of the assay and mean values for all samples at a given time point are shown. (VCA = viral capsid antigen, EA = early antigen). **(B)** SOI was self-assessed by cohort participants at the time points of sample collection. Each individual line represents the SOI score of a single individual over the observation period. The height of grey bars reflects the mean SOI for a given time point. **(C)** A qPCR for *Bam*HI-W repeats was run on 4x10^6^ B cells of cohort participants derived from PBMC samples collected at the indicated time points. Dots represent the results for all available samples. Different colors indicate individual cohort participants. Grey bar graphs indicate the median.

For this study, a cohort of 4 female and 6 male college students with symptoms of IM was enrolled and serum samples were collected over a one-year period. The average age (SD) at time of diagnosis was 22.4 (4.2) years. Acute infection in all participants was confirmed by serology (high viral capsid antigen (VCA)-IgM and undetectable or low Epstein-Barr Nuclear Antigen-1 (EBNA-1) IgG at study entry (Fig 1A (overview), S1 Fig (detailed)). After 12–24 weeks post enrollment (wpe) all cohort participants entered the chronic phase of infection marked by absent or low VCA-IgM, and high VCA-IgG and EBNA-1-IgG titers (7), with three individuals showing serological signs of early virus reactivation (early antigen (EA)-IgG rising after a previous signal decline). At the time of sampling, cohort participants were also asked to self-assess the severity of their illness (SOI) (Fig 1B) using a scoring system that sums the limitations in physical activity and the intensity of pains and symptoms [18]. In line with their serological markers, all participants experienced severe symptoms of mononucleosis during the acute phase of EBV infection (median SOI score at 0 wpe = 4.5/6) but the majority of individuals became asymptomatic within 4 wpe. Further, the frequency of EBV genomes/10^6^ B cells (in the 7 participants with available PBMCs samples or exclusively in cohort participants with positive qPCR signal (n = 6)) seemed to decline over the course of infection (Fig 1C) with a median of 2156 or 2170 EBV genomes at the time of study enrollment, respectively, and EBV reactivation in 3 participants at 12–24 wpe. Sera from 4 EBV-seronegative individuals (EBV-) were included to determine the backround of each respective assay. Furthermore, for all functional assays, sera from 4 chronically EBV-infected individuals (C-EBV+: VCA-IgM^-^ IgG^+^, EA IgG^-^, ENBA-1 IgG1^+^) were included as a reference group.

### EBV proteins induce distinct IgG subclass responses

**Fig 2:**
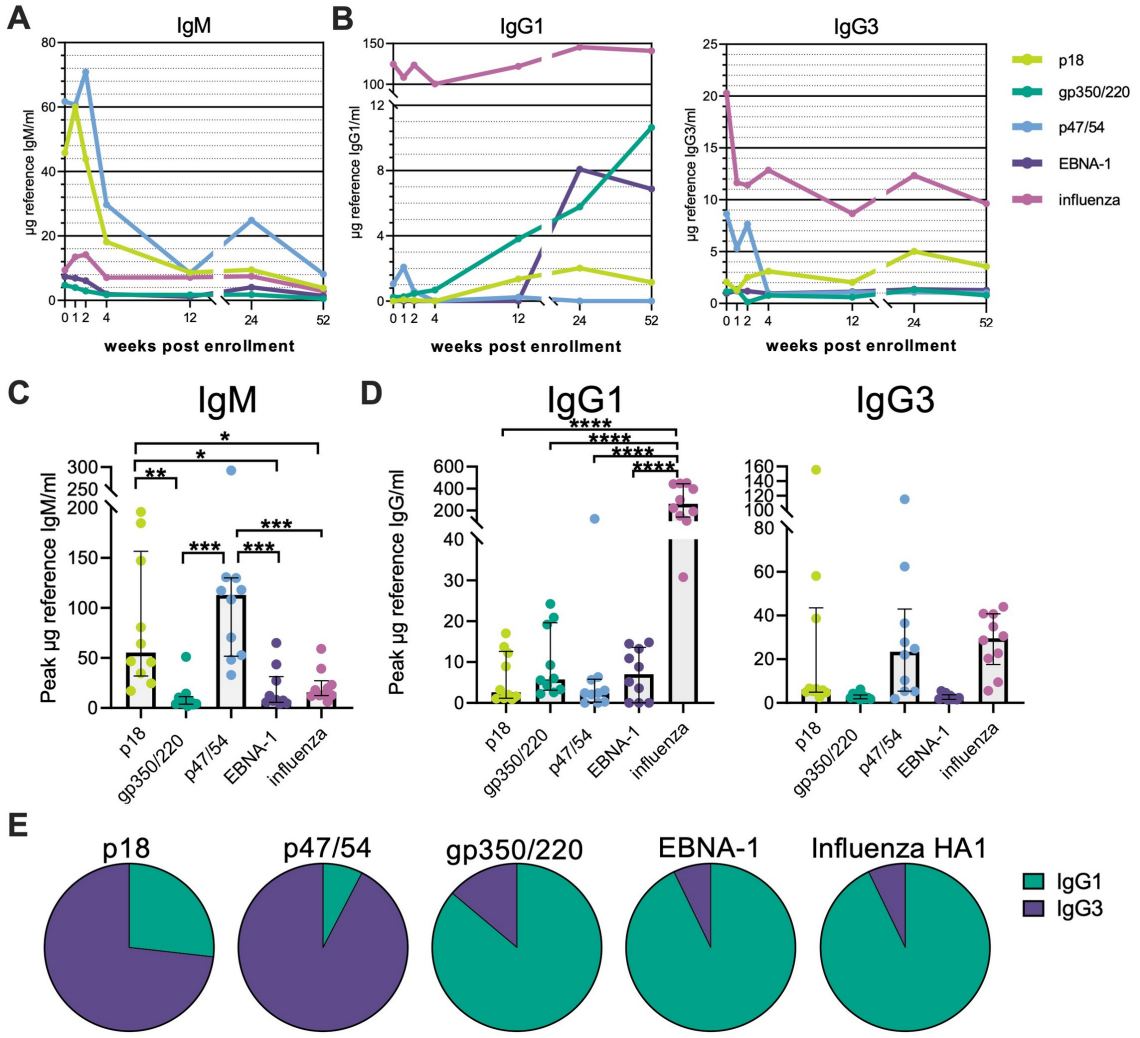
EBV proteins induce distinct IgG subclass responses. **(A)** EBV antigen-specific titers for IgM were determined by class-/subclass-specific direct quantitative ELISAs using a reference Ab of known concentration as standard. Dots represent the median Ab concentration for the indicated time point. **(B)** EBV antigen-specific titers for IgG1 and IgG3 were determined using a similar approach as for IgM. **(C)** The peak median concentration for EBV antigen-specific IgM was calculated. Potential differences between all groups were determined by one-way ANOVA using Tukey`s correction for multiple comparisons. Only statistically significant differences are plotted. **(D)** Peak median concentration for EBV antigen-specific IgG1 and IgG3 were calculated as for IgM. **(E)** Total median IgG1 and IgG3 concentrations over all time points were calculated and accumulative concentrations set as 100% of the respective pie charts.

To dissect EBV-specific Ab trajectories, we quantified antigen-specific IgM responses and the response dominating IgG subclasses IgG1 and IgG3 [14,19]. As antigens we selected capsid antigen p18 (BFRF3), antigen p47/54 (BMRF1) as part of the EA complex, the envelope glycoprotein gp350/220 (BLLF1) as well as the latency protein EBNA-1 (BKRF1). These antigens were chosen as they are the main Ab targets during different stages of the EBV infection cycle and, in the case of gp350/220, have been the target for vaccine efforts [20,21]. Additionally, we included a pool of three influenza HA1s of the 2014–2015 seasonal vaccines as a reference. IgM Abs against all four EBV antigens were induced in the acute phase of disease but rapidly waned (Fig 2A (overview), S2A Fig (detailed)). Beyond 4 wpe, only p18- and p47/54-specific IgM and was consistently detectable (Fig 2A). In accordance with the viral load data suggesting EBV reactivation in some individuals around 24 wpe (Fig 1C), p47/54-specific IgM levels were increased at that time point. Overall, IgM responses were primarily dominated by p18 and p47/54 specificities (Fig 2C; median peak μg reference IgM: p18 = 55.3, p47/54 = 112.8, versus gp350/220 = 5.7, EBNA-1 = 7.7). As previously demonstrated for VCA [19] and gp350/220 [14], EBV infection stimulated the generation of p18-, gp350/220- and EBNA-1-specific IgG1 titers while p47/54-specific IgM failed to switch to a sustained IgG1 memory response (Fig 2B (overview), S2B Fig (detailed)). Nonetheless, EBV-specific IgG1 concentrations were significantly lower compared to influenza HA1-specific Abs, likely as a reflection of the shorter antigen exposure history compared to influenza (Fig 2D). IgG3 responses in contrast were primarily observed for p18 and p47/54 (Fig 2B (overview), S2C Fig (detailed)). In fact, similar to the IgM response, both antigens seemed to dominate this subclass over gp350/220 and EBNA-1 (median peak μg reference IgG3: p18 = 6.1, p47/54 = 23.4, versus gp350/220 = 2.2, EBNA-1 = 2.4, influenza HA1 = 29.6), while the opposite could be observed for IgG1 (Fig 2D). This suggests that lytic and latent EBV proteins possibly induce different IgG subclass profiles (Fig 2E), with IgG3 being the dominating IgG subclass during the active/lytic replication phase and IgG1 dominating the latent antibody response analogous to the influenza HA-1-specific responses.

### Ab-dependent complement deposition (ADCD) during acute infection is exclusively mediated by p18

**Fig 3:**
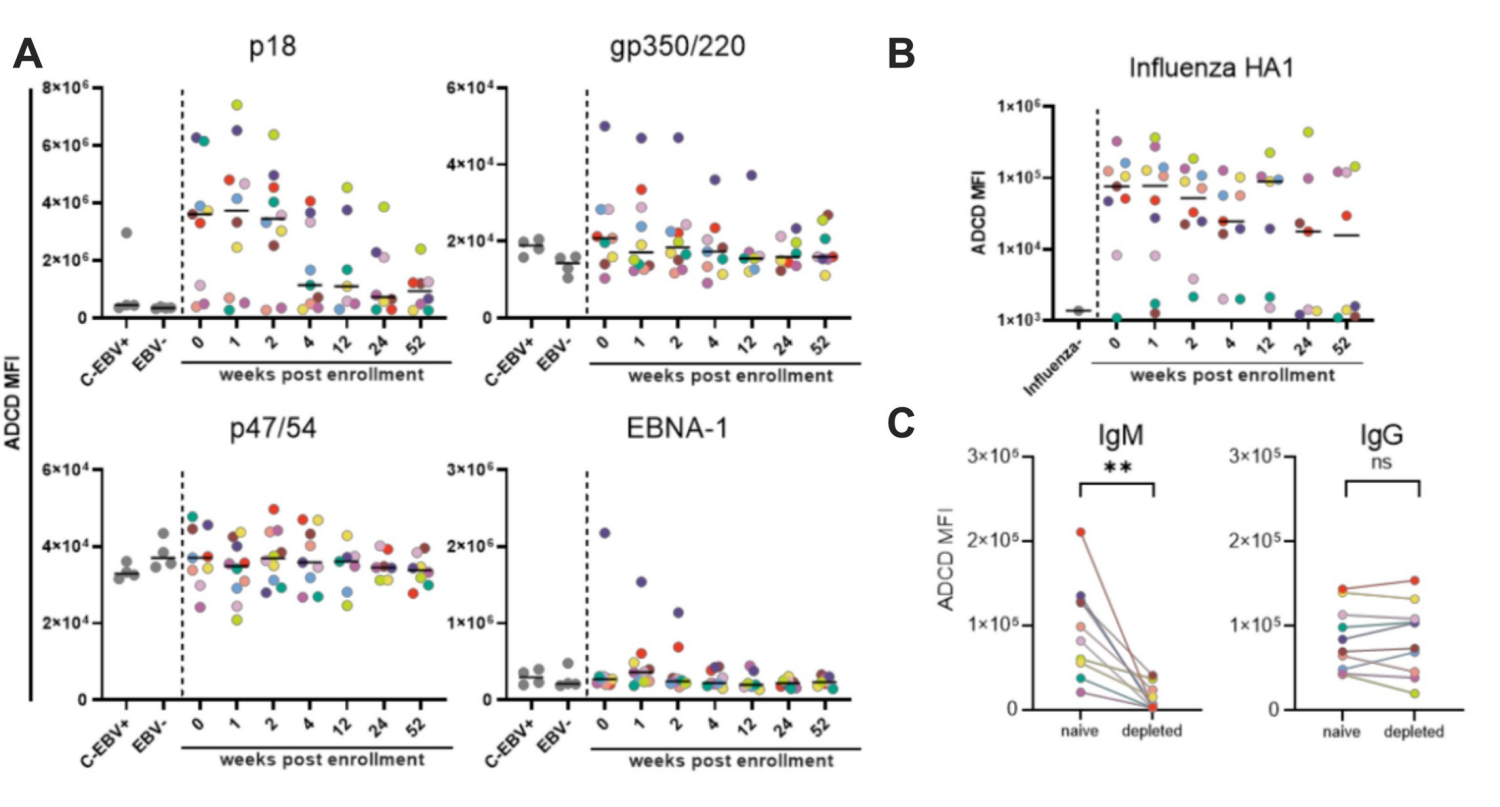
ADCD during acute EBV infection is exclusively mediated by Abs against p18. **(A, B)** The ability of EBV p18, gp350/220, p47/54, EBNA-1- (**A**) and influenza HA1-specific antibodies (**B**) present in cohort samples to mediate ADCD was determined by flow cytometry. Every dot represents the area under the curve of the average measured signal of a given control (grey) or sample. Colors indicate the different cohort subjects and black lines represent the grand median. **(C)** An ADCD assay was performed after depletion of p18-specific IgM or IgG in serum samples of 0 or 1 wpe. Significant differences in the levels of ADCD activity before and after depletion of IgM or IgG were determined by two-tailed T-test.

In order to explore Ab mediated functionality, we first investigated ADCD by measuring the complement deposition on fluorescent beads induced by antigen-specific Abs via flow cytometry (Fig 3A and 3B). Abs against p18 but not against other EBV proteins despite detectable titers induced ADCD in participants (Fig 3A). The response observed for p18-specific Abs was strongest 0–2 wpe (max median MFI: 3.7x10^6^, fold over background: 10.7 at 1 wpe), and decreased rapidly in most individuals thereafter (median MFI/fold over background: 1.1x10^6^/3.3 at 4 wpe, 0.9 x10^6^/2.7 at 52 wpe). No ADCD activity was observed in the reference group of chronically EBV infected individuals (C-EBV+) except for one sample. This suggests that ADCD activity mediated by p18-specific Abs wanes and is eventually lost over time. In contrast, sustained influenza HA1-specific ADCD was observed over the study period indicating the general ability of the hosts to induce Abs with complement depositing activity (Fig 3B). To identify the responsible Ab class for p18-ADCD, depletion studies were performed (Fig 3C), demonstrating that the removal of IgM but not IgG resulted in loss of ADCD activity. Conversely, influenza-specific ADCD in the same individuals mainly depended on IgG (S3A Fig). Thus, IgM mediates p18-ADCD in EBV infection but, consistently with the loss of the p18-IgM response over time, p18-ADCD does not persist into the chronic phase of infection. Overall, this data suggests that EBV infection did not induce ADCD mediating EBV-specific IgG responses.

### Restricted evolution of Ab dependent phagocytosis to p18 during the first year of infection

**Fig 4:**
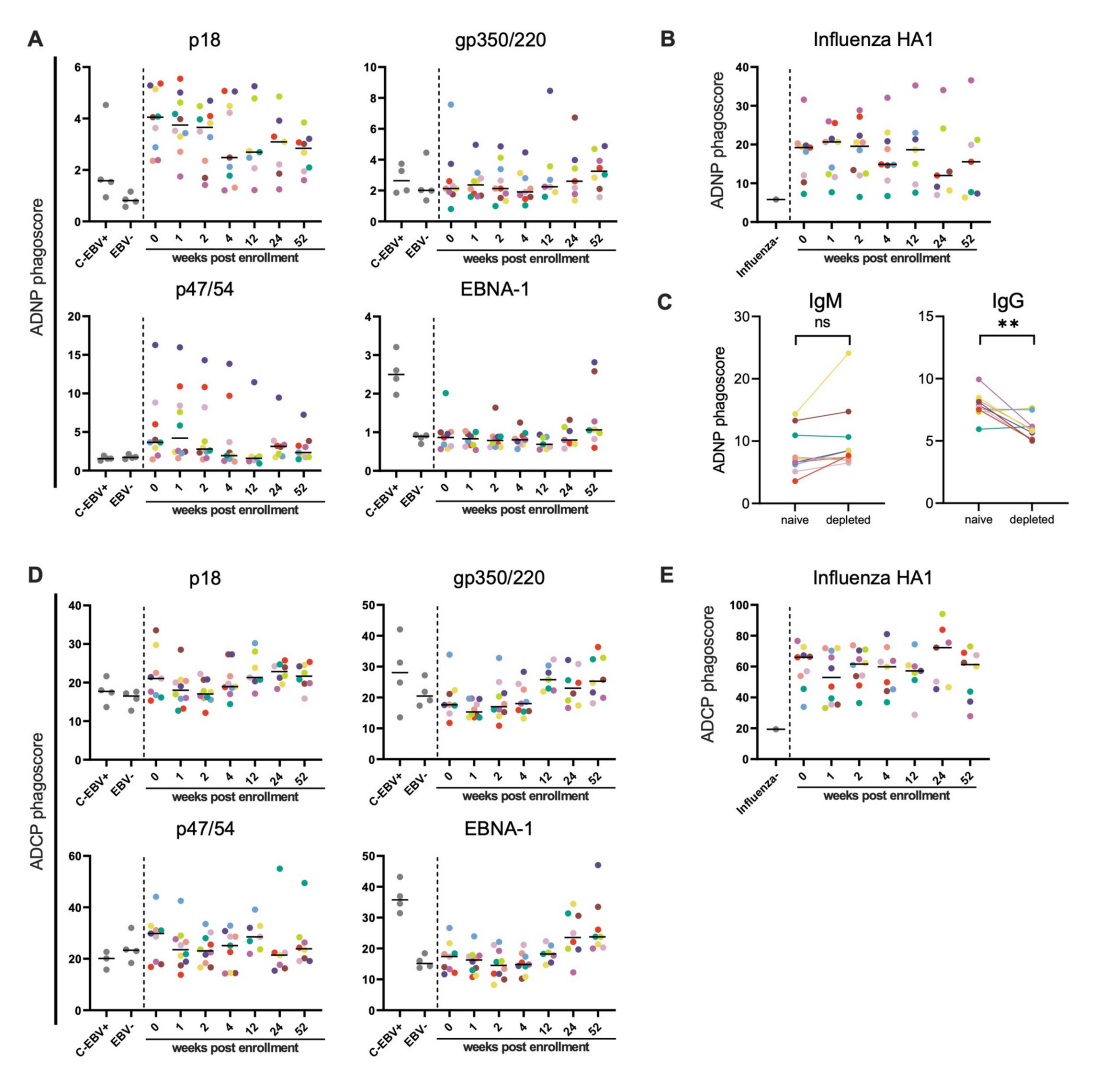
EBV proteins induce Ab dependent phagocytosis inconsistently at low or minimal levels during the first year of infection, except for p18. **(A, B, D, E)** The induction of ADNP (**A, B**) or ADCP (**D, E**) by antibodies against EBV p18, gp350/220, p47/54, EBNA-1 and influenza HA1 was analyzed by flow cytometry. Every dot represents the average measured signal of a given control (grey) or sample. Colors indicate the different cohort subjects and black horizontal lines represent the median. **(C)** An ADNP assay was performed after depletion of p18-specific IgM or IgG in serum samples of 0 or 1 wpe. Significant differences in the levels of ADNP activity before and after depletion of IgM or IgG were determined by two-tailed T-test.

We next investigated the role of ADNP and ADCP (Fig 4A). Most notably, p18-specific Abs induced strong ADNP (median phagoscore/fold over background at 0–2 wpe: 3.7-4/4.5–5) during the early acute phase of infection from 0–2 wpe in all participants (Fig 4A). The levels of reactivity by p18-specific Abs were comparable to influenza-specific Abs (median phagoscore/fold over background at 0–2 wpe: 19.2–20.7/3.3–3.6) (Fig 4B). While in some individuals the p18-ADNP response decreased 4 wpe (median phagoscore/fold over background at 4–52 wpe: 2.5–3.1, 3–3.8), the response overall persisted throughout the observational period in all individuals, and even the C-EBV+ reference group showed residual ADNP activity (median phagoscore/fold over background: 1.58/2). This suggests that p18-specific Abs mediate an ADNP response that lasts at least one year post infection and longer. In the majority of participants p47/54-ADNP was induced from 0–2 wpe but only low p47/54-ADNP activity was detectable at 52 wpe (median phagoscore/fold over background at 0–2 wpe: 2.8–4.2/1.6–2.5; 52 wpe: 2.3/1.4) (Fig 4A). With no reactivity detected for p47/54-specific Abs in the C-EBV+ reference group, together these results indicate p47/54-ADNP is mounted exclusively during the acute and early convalescent phase of EBV infection. Consistent with the rising titer kinetics for gp350/220- and EBNA-1-specific Abs, ADNP activity mediated by these 2 Ab specificities tended to increase at 52 wpe (median phagoscore/fold over background: gp350/220 = 3.3/1.6; EBNA-1 = 1.1/1.2) but only EBNA-1-specific Abs continued to expand ADNP activity over time (median phagoscore/fold over background:2.5/2.8), while gp350/220-ADNP activity stalled (median phagoscore/fold over background: 2.6/1.3). To next identify the Ab class responsible for p18-ADNP, antigen-specific IgM or IgG was depleted from serum samples during early acute infection (0–1 wpe) (Fig 4C). While the depletion of IgM did not result in a reduction of p18-ADNP, the removal of p18- specific IgG significantly decreased p18-ADNP suggesting that p18-ADNP is driven by IgG. Differently, IgM depletion resulted in a significant loss of ADNP function of influenza-specific Abs (S3B Fig).

While neutrophilic phagocytosis was induced, p18 and p47/54 failed to induce similarly robust ADCP (Fig 4D). Instead, activity for gp350/220 and EBNA-1 tended to increase at week 12 or 52 wpe (median phagoscore/fold over background: gp350/220 = 25.8/1.3, EBNA-1 = 23.8/1.6), respectively, (Fig 4D and 4E) and augmented, or at least similar levels of activity were detected in the C-EBV+ reference group (median phagoscore/fold over background gp350/220 = 28.1/1.4, EBNA-1 = 35.8/2.4), consistent with previous reports on ADCP induction by gp350/220 specific Abs [14]. Anti-influenza HA1 Ab-induced ADCP, however, was easily detectable in all participants and frequently at higher levels (Fig 4E).

In summary, while all EBV antigens elicited some phagocytosis mediating Abs, strong and durable phagocytic activity was primarily observed for p18-specific Abs, while beyond one year of infection EBNA-1-specific Abs become the main driver of EBV directed phagocytosis.

### EBV-specific Abs are inefficient at stimulating NK cell degranulation during the first year of infection

**Fig 5:**
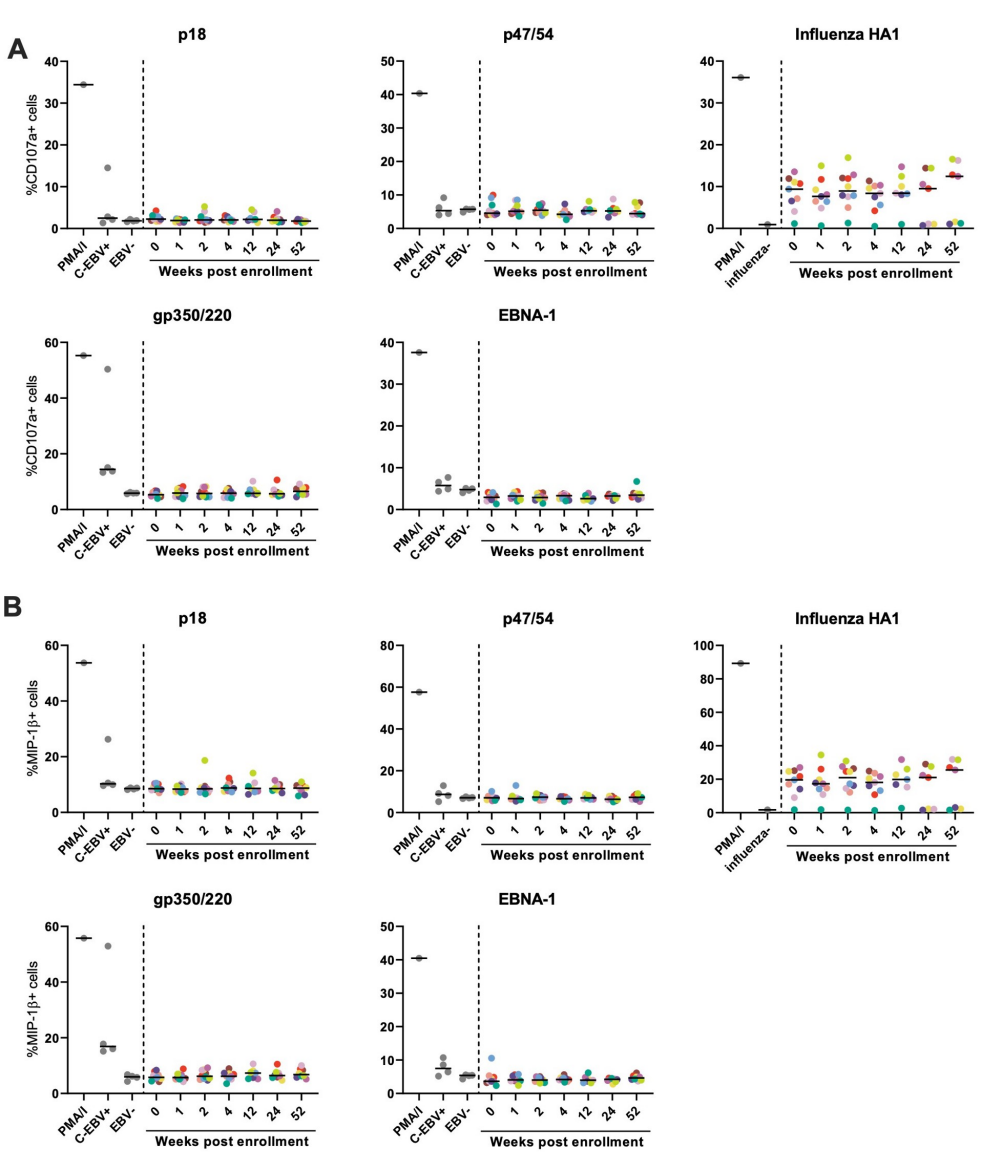
EBV-specific antibodies, including gp350/220 RBD-specific Abs, are inefficient at stimulating NK cell degranulation during acute infection. **(A, B)** EBV antigens p18, p47/54, gp350/220 or EBNA-1 or influenza HA1 as a control were coated to ELISA plates and incubated with serum of EBV infected cohort participants collected at the indicated time points. After an incubation period NK cell degranulation induced by antigen-specific antibodies or PMA/I as an unspecific control was assessed by determining the percentage of CD107a (**A**) or MIP-1beta (**B**) positive NK cells by flow cytometry. Every dot represents the average measured signal of a given control (grey) or sample. Colors indicate the different cohort subjects and black horizontal lines represent the median.

Previous work demonstrated that in the chronic phase of EBV infection Abs directed against gp350/220 can mediate NK cell degranulation, cytokine production, and ADCC [12,13,22]. To investigate the ability of EBV-specific Abs against p18, p47/54 and EBNA-1 in addition to gp350/220 to mediate NK cell function over the course of infection, NK cell degranulation was detected by measurement of CD107a (Fig 5A) and MIP-1β (Fig 5B) expression post immune complex stimulation. Robust NK cell degranulation and MIP-1β secretion was stimulated by treatment of NK cells with phorbol myristate acetate/ionomycin (PMA/I), which served as an unspecific positive control. Similarly, influenza HA-1-specific Abs in the serum samples of nearly all participants induced NK reactivity. In contrast, EBV-specific Abs of our cohort induced minimal NK functionality with only selected individuals demonstrating NK-cell degranulation in response to p47/54-, p18- and gp350/220-specific Abs during the first year of infection. Only gp350/220-specific Abs in samples of the C-EBV+ reference group induced CD107 (%+ cells: 14.4, fold over background: 2.4) and MIP-1β expression (%+ cells: 16.9, fold over background: 2.8), however, these levels remained below the activities observed by HA-1-specific Abs. These findings indicate that EBV-Ab mediated NK activation is only detected at low levels in the chronic phase more than one year after infection.

### EBV p18-specific Abs bind highly efficiently to FcgRIIIb

**Fig 6:**
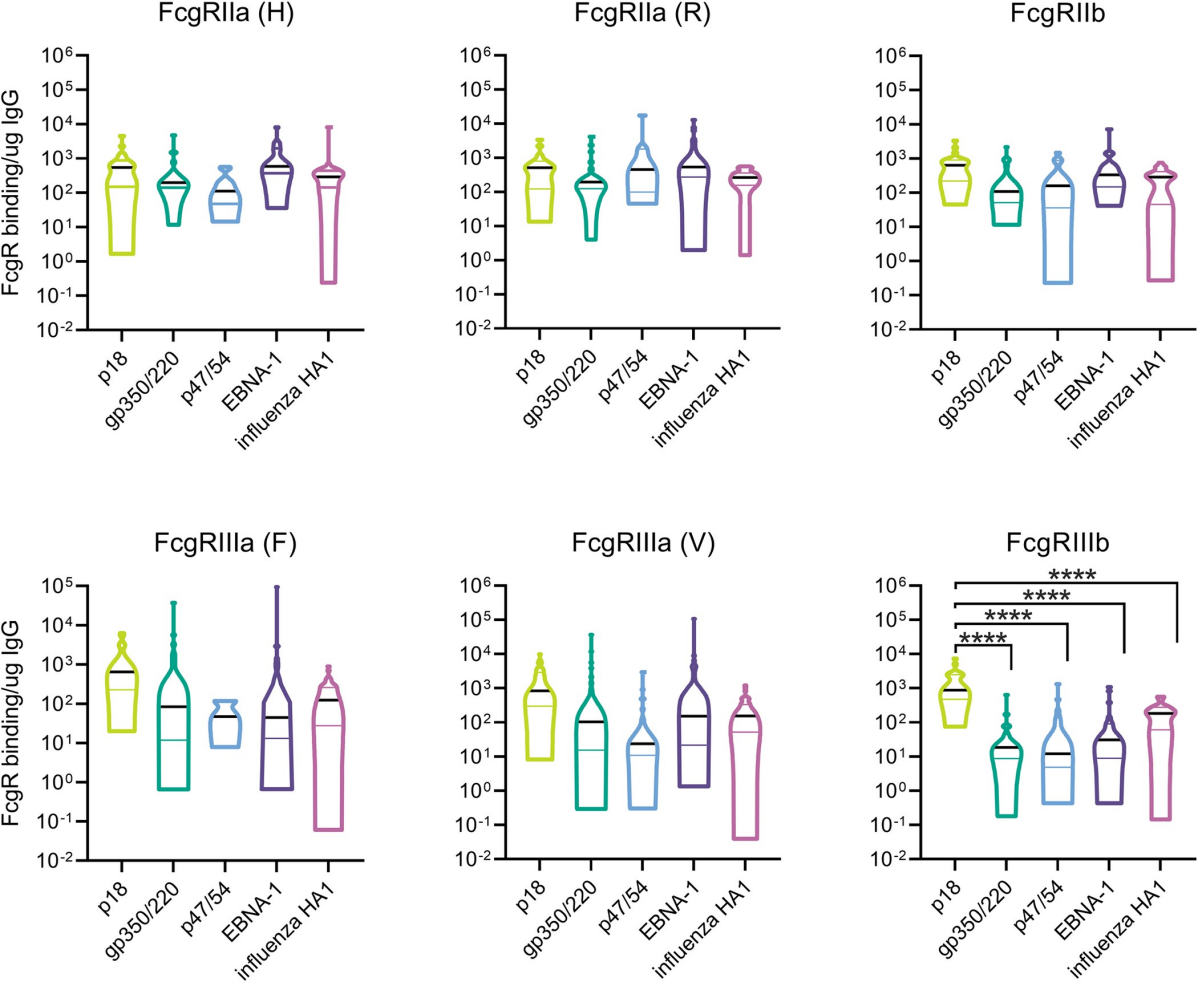
EBV p18-specific Abs bind highly efficiently to FcgRIIIb. The ability of antigen-specific EBV and influenza antibodies to bind to FcgRs was measured by luminex. To account for titer dependent influences on FcgR binding signals, for each sample the ratio between FcgR binding signal and the sum of IgG1 and IgG3 titers totaling to above 0.1μg/ml was calculated. Lines in violin plots represent the divisions between quartiles, with the black line representing the grand median. A one-way ANOVA with Tukey’s correction for multiple comparisons was applied to determine statistical differences between all groups. Only statistically significant differences are plotted.

To assess the potential influence of FcgR binding efficiency of EBV-specific Abs on Fc-mediated functionality, the ability of p18-, gp350/220-, p47/54- and EBNA-1-specific Abs to interact with FcgRs was assessed by multiplex flow cytometry (Fig 6). After normalizing the data to total IgG1 and IgG3 concentrations above 1μg/ml to account for titer dependent differences, binding of EBV-specific Abs to FcgRs was mostly comparable across the different EBV antigens and similar to the binding pattern of influenza HA1-specific Abs. However, p18-specific Abs bound significantly better to FcgRIIIB than Abs against influenza or other EBV proteins (median signals: p18 = 875, gp350/220 = 19, p47/54 = 12, EBNA-1 = 31, influenza HA1 = 183). The same trend was observed for both genotypes of FcgRIIIA (158F, 158V) that were analyzed. These data suggest that FcgRIIIB could play a role for ADNP mediated by p18- specific Abs. Moreover, these data indicate that the relatively low Fc-functionalities mediated by the assessed EBV-specific Abs were not due to insufficient Fc-receptor binding.

### The breadth of EBV-specific Ab responses expands from acute to chronic infection

**Fig 7:**
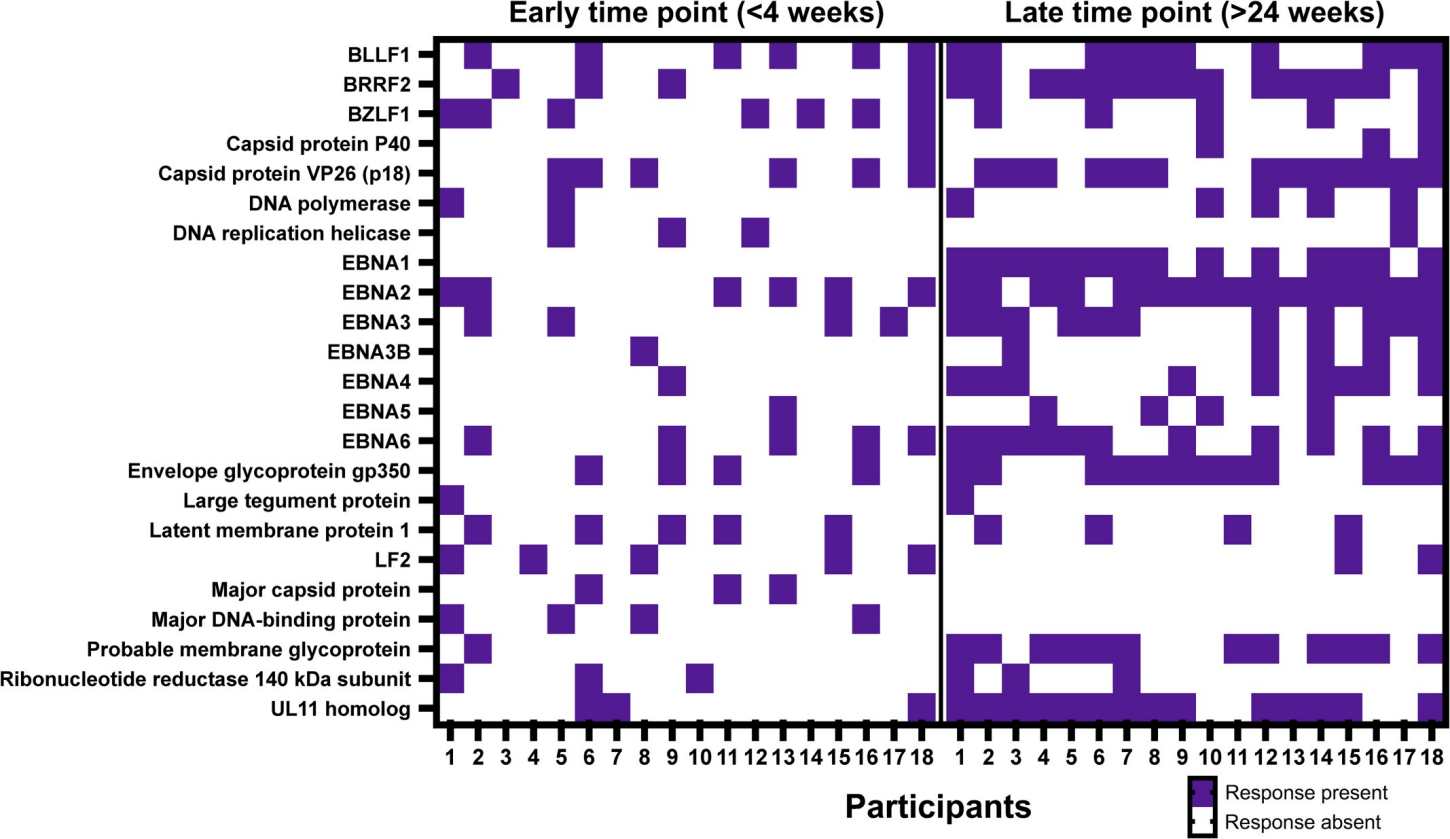
The breadth of EBV-specific Ab responses expands from acute to chronic infection. IgG recognition of EBV-specific peptides was determined in 18 individuals at an early (<4 wpe) and a late (>24 wpe) time point of EBV infection by the phage-based VirScan assay. The heatmap shows antibody responses to EBV proteins when antibody binding was detectable in at least two individuals.

Although all participants were enrolled with acute IM suggesting robust immune activation, we observed a quantitatively and qualitatively rather inefficient induction of Fc-functional Abs against the 4 selected EBV antigens. We therefore proceeded to map the total breadth of the EBV-specific humoral responses in acute and chronic infection using the Phage Immunoprecipitation sequencing (PhIP-seq) based VirScan assay [17]. This assay uses phage-display library overlapping 56mer peptides of more than 1,500 different pathogens including 2,263 peptides from six different EBV strains. We measured IgG Ab responses for an earlier timepoint (<4 wpe) and a later time point (24–52 wpe) in all 10 individuals but also included 8 additional participants (7 male/1 female, average age (SD) = 20 years (2.3)) from our cohort for this analysis. Overall, the breadth of responses during acute infection was heterogenous and response pattern differed between individuals (Fig 7). As expected, the breadth of responses was larger at the later time point, specifically selected antigens such as EBNA-1/EBNA-2 and gp350 were recognized by most of the participants. Responses against respiratory syncytial virus (RSV), influenza or rhinoviruses were readily detectable (S4 Fig) and persisted at comparable levels between both time points (Wilcoxon matched pairs signed rank test, p = 0.053, p = 0.28 and p = 0.13 for RSV, influenza and rhinovirus A, respectively). This unbiased approach therefore confirmed that the observed deficiency of EBV to induce robust and broad Fc-functionality across antigen-specificities is not a consequence of an overall inability of EBV to induce a broadening humoral immune response.

### EBV-specific IgM and Fc-functions mediated by p18-specific Abs correlate with SOI

Given the restricted number of antibody responses observed in acute EBV, we next aimed to define whether any specific antibody-function was associated with control of symptomatology. In fact an inverse relationship between gp350/220 Ab titers and the SOI score in a cohort of more than 300 participants [23] has been reported previously. To assess whether any of the measured Ab-associated determinants in our analysis was linked to SOI, all collected data was grouped depending on the self-reported SOI at the times of sample collection. SOI values of 0–1 were considered as low and values of 2–6 as high. Using this approach, a positive relationship between titers of EBV-specific IgM and SOI was identified (S5 Fig; median values low vs. high SOI: p18 = 10.15/40.73, gp350/220 = 1.52/4.16, p47/54 = 17.22/44.68, EBNA-1 = 1.56/5.72). In addition, a high self-reported SOI was linked to significantly increased levels of p18-Ab mediated ADNP and ADCD (S5 Fig; median values low vs. high SOI: ADNP = 1.91/2.90, ADCD = 0.9x10^6^/3.2 x10^6^) suggesting a potential role of these Fc-mediated functions in disease modification during EBV infection.

### Acute EBV infection fails to induce a persistent Fc-functional Ab profile compared to acute HIV infection

**Fig 8:**
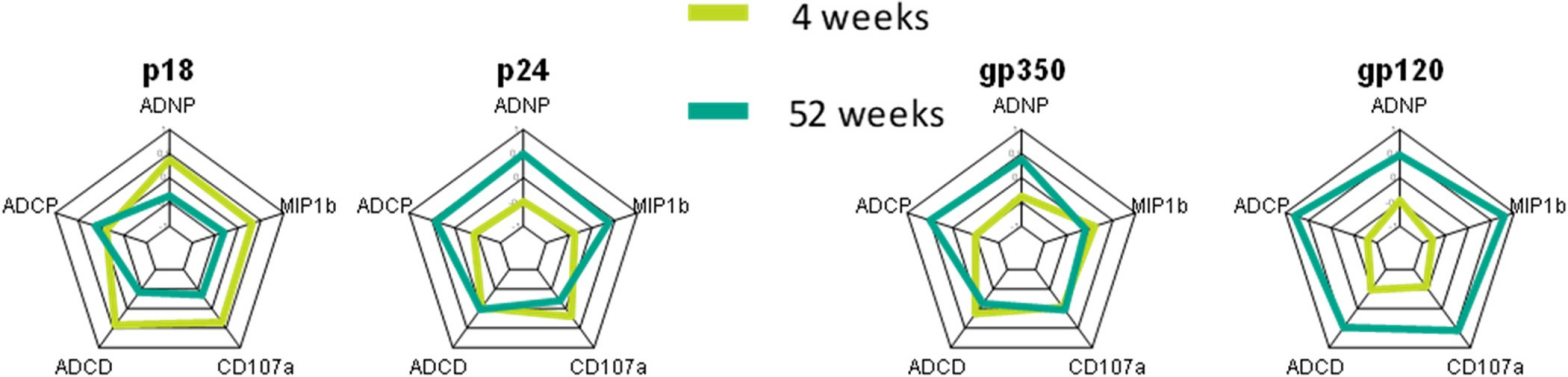
Acute EBV infection fails to induce a persistent Fc-functional Ab profile compared to acute HIV infection. Radar plots show the average value of the Z-scored functional data for EBV and HIV antigens (in the respective cohort) at the 4 or 52-week time point.

Previous data point to robust and rapid induction of functional humoral immune responses in other viral infections [24,25], including HIV [26]. Thus to contrast the distinct EBV profiles observed here to HIV, we next compared the functional Ab profiles during acute HIV infection with our acute EBV infection Ab signatures using a previously acquired dataset [26]. Specifically, we focused the comparison on two early and late antigens, the HIV p24 and the EBV p18 as early Ab targets, and the HIV envelope gp120 and the EBV envelope gp350/220 as late Ab targets. When we compared the functions, p18 induced a functional profile early that contracted during the later time point while p24 induced an expanding profile with increased functionality at the later time point (Fig 8). Similarly, the gp350/220-specific response remained relatively unchanged between the acute and the late time points with only some increase in ADCP and ADNP activity while the gp120 response demonstrated robust expansion of all Fc-functions over time. These data therefore suggest that while both infections induce functional Ab responses, these only expand over time in HIV infection while functional Ab levels in EBV infection contract.

## Discussion

While previously thought to be largely innocuous, emerging data clearly highlight the pathological role of EBV infection in driving autoimmunity (reviewed in [27]) and malignancies [28,29] in a small, but not insignificant portion of the population. Thus, vaccines and other immunotherapies are urgently needed to temper the pathology caused by this virus. While dogmatically, the field hypothesized that neutralizing Abs could confer protection against the virus, the vaccine-induction of robust levels of gp350-specific neutralizing Abs did not protect against infection [20]. Thus here we aimed to define potential additional humoral mechanisms associated with viral control, beyond neutralization, by systematically focusing on Ab Fc-functional activities during acute to convalescent EBV infection.

We found the classical serological pattern to the early and latent antigens that are characteristic for EBV infection observing a robust initial IgM response to EBV lytic antigens tested, but not to EBNA-1. However, we noticed two developing pattern of IgG subclass response profiles separating the early/lytic antigens that induced more IgG3 from the latent/convalescent antigens that elicited primarily IgG1. As IgG3 is the most polyfunctional of the IgG subclasses and is known to mediate especially potent Fc effector functions [30], the preferential induction of this subclass by the early/lytic antigens potentially suggests their role in direct antiviral activity, such as controlling the infection. Our sample size, however, was too small to assess a potential correlation between EBV-specific IgG3 responses and EBV genomes counts and kinetics. In general, IgG titers against any of the early but also against the latent antigens did not reach the levels of influenza-specific Abs within the same hosts despite the participants having IM with substantial symptomology suggesting robust immune activation [18]. Using VirScan we were able to comprehensively map the antibody response landscape confirming that the responses broadened over the duration of study follow-up, suggesting sufficient antigen exposure and appropriate induction of at least binding Abs.

While the titers of EBV Abs were low compared to influenza-specific responses, these Abs were capable of mediating a variety of Fc-functions. Specifically, p18 clearly induced IgM-driven ADCD in the acute phase of infection. So far, it had not been determined whether EBV also interacts with the classical complement system by inducing ADCD. In fact, EBV infects B cells via gp350/220 interaction with CR1/2 [31–33], which are regulators of the complement system. Furthermore, gp350/220 activates C3 deposition via the alternative complement pathway (ACP) [34] and the decay of ACP C3 convertase is accelerated by EBV through an unknown mechanism [35]. Our data therefore describe an additional pathway of Ab mediated complement activation during EBV infection. IgM is known to have the greatest potential to fix complement, followed by IgG3 and IgG1 [36] and depletion of p18-specific IgM indeed abrogated the ADCD activity of participants sera. Overall, ADCD serum activity declined over time in the presence of rising titers of gp350/220 and EBNA-1-specific Abs, suggesting that Abs to the latter antigens were not equipped to mediate complement activation via Fc-mechanisms.

Both p47/54- and p18-specific Abs across the majority of participants mediated ADNP during the acute phase of infection. Specifically, neutrophil activity mediating p18-specific Abs persisted into the chronic phase of infection, therefore representing the only EBV-specific Fc-functional Ab response that was permanently induced in the first year of infection. Furthermore, p18-ADNP activity was comparable to ADNP mediated by influenza HA1-specific Abs which were present in the samples at much higher concentrations suggesting that p18-specific Abs are particularly efficient at triggering ADNP. In the chronic phase of infection at least one year post infection, Abs against latent proteins including EBNA-1 and gp350/220 dominate neutrophil activation against EBV.

FcgR binding studies confirmed that p18-specific IgG bound with increased efficiency to FcgRIIIB and a similar trend was observed for FcgRIIIA compared to the other Abs investigated in this study suggesting that these FcgRs might be critical for p18-specific Abs to induce ADNP. One hypothesis is that p18-specific Abs Fc are less fucosylated, as this has been associated with enhanced binding to FcgRIIIa and FcgRIIIB [37,38], however one could have expected more NK-cell inducing activity in this case. The strong binding of p18-specific Abs to FcgRIIIB further suggests that ADNP is induced by FcgRIIIB recruitment of p18-specific Abs to FcgRIIa [39,40]. Alternatively, it is theoretically also possible that FcgRIIIB transfers p18-specific Abs to FcgRIIIA on neutrophils, which was recently described to be expressed at low but sufficient levels to mediate potent ADNP [41]. Although the p18-ADNP response correlated with disease severity, it remains uncertain if such responses were purely a reaction to significant immune activation in the setting of severe IM or if the response was induced to actively contribute to viral control. A similar association had been previously reported between the magnitude of cytotoxic CD8+ T-cells and disease severity [42].

We also observed gp350/220 and EBNA-1-Ab mediated ADCP, as suggested by the FcgR binding study of antigen-specific Abs, but these responses were much lower than influenza HA1-specific antibodies mediated activity within the same hosts. Only in the C-EBV+ group we observed increased ADCP activity suggesting that the EBNA-1-specific response further matures over time.

Similarly, NK cell degranulation as a measure of NK-inducing Ab activity, was substantially decreased during the first year of infection compared to what had been previously described by others [12,13]. However, in line with previously published observations, gp350/220-specific Abs in individuals with chronic EBV infection were capable of engaging NK cell functions [43,44], although not as efficient as influenza HA1-specific antibodies. Therefore, the non-neutralizing Ab response to EBV is characterized by an immediate induction of ADCD and ADNP by p18-specific Abs during the acute phase of infection. While ADCD activity is lost during the first year of infection, ADNP functionality persists beyond that time frame. Induction of ADCP and NK cell functions are only developing slowly, at least one year post infection, and are dominated by latent EBV antigens.

While accumulating evidence exists on the ability of emerging viruses to induce a variety of non-neutralizing, but Fc-functional Ab responses [24–26], EBV failed to induce a similarly robust functional Ab response. One likely explanation is persistent antigenemia that is present for example during HIV-1 infection but absent for most individuals during EBV infection. Moreover, the timing of release of EBV antigens is distinctly linked to the viral life cycle starting with the release of p47/54 DNA-replication complexes as "stable" particulate structures from apoptotic cells as well as the release of incomplete VCA-p18 both inducing strong IgM responses during the early lytic stage. This is followed by limited intact virion release resulting in less robust gp350 B-cell receptor engagement and upon transition from lytic stage to latency, waning of early antigens and virion/capsid release. Consequently, IgM induction decreases while strong T-cell responses lead to apoptotic EBNA-1-DNA complex release and IgG induction [7]. During each phase of the EBV life cycle, the degree of antigen-exposure might therefore be sufficient to induce binding Abs but seems to be inadequate to effectively trigger Ab Fc-functional enhancement. One can only speculate that EBV utilizes this phase-specific antigen presentation as a mechanism of immune-evasion, preventing the induction of lasting and highly Fc-functional Abs, similar to what has been described for EBV medicated evasion from cytotoxic CD8+ T-cell responses [45]. Immune-evasion has also been proposed as a risk factor for EBV-associated hematological malignancies such as HL, however, it remains to be determined if Fc-functional antibodies play a role in oncogenesis and if the lack or loss of such antibody functions might fascilitate malignant transformation. Additional studies to further evaluate these questions are needed. In summary, using our comprehensive Ab profiling approach, we were able to systematically describe the evolution of Fc-functional EBV-Ab responses during the first year of infection and in response to changing antigen targets during the EBV life cycle. The levels of both Abs and Fc-functional activity however were surprisingly limited despite significant symptomatic disease and differed from other virus-specific Abs against influenza or HIV. Future studies should explore if the existent Ab signatures can be further augmented and/or enhanced via immune therapeutic interventions such as vaccination.

## Materials and methods

### Ethics statement

The collection of all human serum samples in this study was conducted in accordance with the Declaration of Helsinki and the acute EBV infection cohort study was approved by the MIT Committee on the Use of Humans as Experimental Subjects (COUHES) (approval #2010P002463). Primary cells from healthy human donors were isolated from fresh blood or buffy coats collected at MGH under approval by the Partners Institutional Review Board (approval #2005P001218). All subjects provided informed written consent.

### Study design

The aim of this descriptive study was to provide an in-depth characterization of the specificities and Fc-functionality of anti-EBV Abs from the acute to chronic stage of infection. Viral antigens EBV capsid protein p18, envelope protein gp350/220, early protein p47/54 and latent protein EBNA-1 were selected based on their known ability to induce Ab responses in the majority of individuals [7]. Due to their consistency Ab responses against p18-, p47/54- and EBNA-1 are utilized in standard EBV diagnostics, while gp350/220-specific Ab responses were found to be significant for the prevention of IM after vaccination [20]. To provide a reference, we also characterized Ab responses to influenza HA1s of the 2014–15 vaccine formulation.

All experiments in the study were conducted using samples of 10 American college students, with the exception of VirScan analysis for which the continued recruitment of study participants allowed us to include samples of an additional 8 individuals. All donors were recruited with confirmed diagnosis of IM and sampled up to 7 times during the first year of enrollment (total number of samples for the first 10 donors: 61) following the evolution of their Ab responses from the acute to chronic phase. Cohort size selection was based on the availability of samples at the time.

Ideal antigen coating concentrations and sample dilutions within linear range were ensured for all flow cytometry assays, including luminex-based techniques. We further performed in-depth method optimization experiments to generate the protocols for in-house ELISAs and the adapted qPCR technique. In all experiments, a single technical repeat was measured with the exemption of ELISAs to determine reference Ab titers and VirScan analysis, which were performed using duplicates. At least two biological repeats were performed for all experiments with the exception of data generated by standard diagnostic ELISA (IVD certified), qPCR (limited sample availability) and VirScan (costs of technique). Outliers were excluded based on known human or technical error.

### Human subjects

The study enrolled adult males and females ages 18–50 years with a clinical syndrome consistent with infectious mononucleosis (including but not limited to sore throat, malaise, fatigue, fever, pharyngitis, enlarged glands, difficulty swallowing, altered sleep), and positive EBV serology consistent with early infection defined as a positive anti-VCA (IgM or IgG) Ab and a negative EBNA-1 IgG Ab. Subjects on immunomodulatory medications, with history of immune immunodeficiency syndromes including HIV-infection, history of splenectomy/thymectomy, history of bone marrow or other organ transplantation, and pregnancy at time of recruitment were excluded. Serum and PBMCs were collected at MIT’s Clinical Research Center from study participants at enrollment, week 1, week 2, month 1, month 3, month 6, and month 12 post enrollment. The cohort that was analyzed for HIV-specific humoral immune responses has been described in detail in Jennewein et al. [26].

### Primary cells

For the isolation of primary human neutrophils from healthy donor blood leucocytes were separated from erythrocytes by mixing the blood in a 5:1 ratio with Hetasep (Stemcell Technologies). After an incubation of 15 min at 37°C the leucocytes were washed (500xg, 10 min, 4°C) with MACS buffer (PBS, pH 7.2 + 0.5% BSA + 2mM EDTA, sterile filtered) and purified using the direct human neutrophil isolation kit (Stemcell Technologies). Purified neutrophils were suspended in R10 at density of 2.5x10^5^ cells/ml and used immediately for experiments. Human B cells from individuals of the EBV-infected cohort were isolated from frozen PBMCs after DNAseI treatment by negative selection using the EasySep human B cell isolation kit (Stemcell Technologies).

### Cell lines

All cell lines were obtained from the NIH AIDS Reagent Program, grown in a humidified incubator with 5% CO_2_ at 37°C and were not authenticated before use. The THP-1 cell line is a monocyte cell line derived from a male infant with acute monocytic leukemia. These cells were cultured in R10 with 0.05mM 2-mercaptoethanol and culture densities were kept below 5x10^5^ cells/ml to maintain consistent levels of FcgR expression.

### Antigens and Ab controls

Large multi-epitope peptides (50–64 AA’s in length) representing immunodominant regions of p18 (BFRF3), p47/54 (BMRF1) and EBNA-1 (BKRF1) (sequences proprietary) were provided by J.M. Middeldorp. The receptor binding domain (RBD) of EBV gp350/220 (IT-005-034p, aa4-450) as well as Influenza HA1s of the 2014–2015 vaccine formulation (IT-003-SW12p, IT-003-00427p, IT-003-B19p) were purchased from Immune Technologies. Influenza antigens were used as a mix (1:1:1 ratio). Serum of 4 individuals with chronic EBV infection (VCA IgM^-^ IgG^+^, EA IgG^-^, EBNA-1 IgG^+^) (Plasma Services Group) was included as a reference, and influenza-, (MBL International) or EBV-negative serum (Plasma Services Group) served as a negative control to assess assay background signals.

### SOI assessment

SOI was self-evaluated by cohort participants at every study visit utilizing a previously published assessment questionnaire [18]. In brief, both physical limitation and symptom/pain intensity was evaluated on a scale from 0 (absent) to 3 (severe interference with daily activities) and the results summed up to generate a final SOI score.

### ELISAs

The presence and state of EBV infection in cohort subjects was determined by LabCorp using CLIA approved diagnostic ELISAs measuring the Ab response to viral VCA, early antigen and EBNA-1. Disease states were classified as following: acute primary infection (VCA IgM^+^, VCA IgG^-/low^, EBNA-1 IgG^-^), latent infection (VCA IgM^-/low^, VCA IgG^+^, EBNA-1 IgG^+^) and reactivation of infection (VCA IgM^+^, VCA IgG^+^, EBNA-1 IgG^+^).

Antigen-specific Ab titers in serum samples were measured in direct ELISAs with Ab-class/subclass-specific Ab-coupled HRPs (IgM: clone HP6083, Invitrogen; IgG1: clone HP6069, Thermo Fisher; IgG3: clone HP6047, Thermo Fisher). Due to the lack of available matched pairs of antigen-specific Abs, a standard for the determination of Ab titers was generated by direct coating of Abs of the class/subclass of interest (Sigma) on the plate in known concentrations. Binding of secondary Abs was detected by measurement of light absorption (wavelength 450nm-reference wavelength 570nm) using an Infinite M1000 Pro (Tecan) plate reader.

### Flow cytometry

To investigate Ab effector functions previously published flow cytometry assays of the systems serology platform [16] were used. In brief, ADCP [46] and ADNP [47] was assessed using yellow-green fluorescent, NeutrAvidin-coated microspheres (Invitrogen), which were coupled in a 1:1 ratio (μl/μg) overnight at 4°C with biotinylated antigen. The antigen-coupled beads were incubated with 1-2hs at 37°C with serum samples diluted in PBS, washed, and incubated with THP-1 cells or primary human neutrophils for 16 or 1 hour, respectively. Neutrophils were stained with CD66b-Pacblue (clone G10F5, Biolegend) and both THP-1s and neutrophils were fixed with 2% PFA prior to detection of phagocytosis by flow cytometer. A phagoscore was calculated using the following formula to express the efficiency of bead uptake: geometric mean of bead^+^ cells x %bead^+^ cells of total cell population /10,000.

For the analysis of ADCD [48], red fluorescent Neutravidin-coated microspheres (Invitrogen) were coated as described for the phagocytosis assays. Complement present in serum samples was inactivated for 30min at 56°C and aggregates pelleted by centrifugation for 5min at 21,000xg. Three dilutions of each serum sample in RPMI-1640 were incubated with beads for 15min at 37°C. Freshly reconstituted untreated or, as a negative control, heat-inactivated guinea pig complement (Cedarlane) was diluted 1:60 in veronal buffer with 0.1% gelatin (Boston Bioproducts), added to the wells and incubated for 20min at 37°C. Afterwards, beads were washed 3x with PBS with 15mM EDTA by centrifugation at 1000xg and stained for 15min in the dark with FITC-Conjugated Goat IgG Fraction to Guinea Pig Complement C3 (MP Biomedicals). Finally, beads were washed 3x with PBS and complement deposition was measured immediately thereafter. Antibody mediated degranulation of NK cells was described previously [49]. Briefly, 96-well ELISA plates (Nunc) were coated with 2ug/ml antigen overnight at 4°C and blocked under the same conditions with 5% BSA in PBS. Serum samples at a dilution of 1:100 or PBS were added to the wells and the plates incubated at 37°C for 2hs. Per well, 5x10^4^ primary human NK cells in R10 stained were together with 5mg/ml brefeldin A (Sigma), GolgiStop (BD) and anti-CD107a-PE-Cy5 (clone H4A3, BD). As an unspecific positive control, phorbol 12-myristate 13-acetate and ionomycin (PMA/I) was added to wells containing NK cells and PBS. The plates were incubated for 5h at 37°C and the cells fixed and permeabilized using Perm A/B (Life Technologies). NK cells were stained with anti CD3-A700 (clone UCHT1), anti-CD16 APC-Cy7 (clone 3G8), anti-CD56 PE-Cy7 (clone B159) and anti-MIP-1β-PE (clone D21-1351) (all BD). Results of the flow cytometry assays were measured using flow cytometers of the models LSRII (BD), iQue Screener Plus (Intellicyt) or S1300EXi (Stratedigm).

### Depletion of antigen-specific Abs

IgG or IgM were depleted from serum samples using Protein A/G agarose plus (Thermo Scientific Pierce) or IgM CaptureSelect matrix (Thermo Scientific), respectively. The matrices were washed 5x with PBS and incubated with samples diluted 1:5 in PBS. After incubation overnight at 4°C with agitation the depleted flow through was recovered. To increase the yield, the matrices were washed with one sample volume PBS and flow throughs pooled with the depleted samples resulting in a total 1:10 dilution of the depleted samples. Non-depleted samples were treated in the same way without depletion matrix. IgM and IgG depletion were confirmed by ELISA.

### Luminex assays

The ability of antigen-specific Abs to bind to FcgRs was assessed using Luminex technology following published protocols [50]. In brief, magnetic carboxylated fluorescent beads (Luminex Corp.) of different regions were coupled to biotinylated antigens in a two-step carbodiimide reaction. First, beads were washed in PBS with 0.05% Tween-20 and activated for 30 min in 100mM monobasic sodium phosphate, pH 6.2, with freshly added 5 mg/ml of Sulfo-NHS and EDC (both Pierce). Subsequently, beads were washed in 50 mM MES, pH 5.0, and incubated with antigen (antigen:bead ratio in w/v = 12.5%) for 2h on a rotational mixer. Antigen-coupled beads were washed and blocked for 30 min in PBS-TBN (PBS-1×, 0.1% BSA, 0.02% Tween 20, 0.05% sodium azide, pH 7.4), incubated with serum samples overnight at 4°C under shaking, and washed. To measure FcgR binding, biotinylated FcgR receptors (Duke Human Vaccine Institute) were coupled to streptavidin-PE (Phycolink), quenched in 5mM biotin (Avidity) and used for detection. All assays were measured on a BioFlex 3D (Bio-Rad).

### qPCR

To determine the number of EBV infected B cells present in cohort subjects, qPCR measuring the number of EBV BamHI-W repeats was detected using synthesized W repeat DNA as a standard (Integrated DNA Technologies). Genomic DNA was isolated from 4x10^6^ B cells using the QIAamp DNA Mini Kit (QIAGEN), dried by vacuum centrifugation and dissolved in PCR-grade water to a concentration of 100μg/ml. Per reaction 4ul sample were mixed with 2ul forward (5’-3’ AGTGGGCTTGTTTGTGACTTCA) and reverse primer (5’-3’ GGACTCCTGGCGCTCTGAT) (final concentrations 250nM), 2 ul FAM-Zen/Iowa Black FQ-labeled probe (5’-3’ TTACGTAAGCCAGACAGCAGCCAATTGTC) (final concentration 250nM, Integrated DNA Technologies) and 10 ul iQ supermix (Bio-Rad). For sample quantification on a LightCycler 480 (Roche), an initial PCR cycle of 3min at 95°C was run, followed by 50 repeats of second cycle (15s at 95°C, then 1min 60°C).

### Pathogen-specific IgG repertoire analysis (VirScan)

VirScan analysis was performed and analyzed as described before [51]. In brief, serum samples were incubated with VirScan 3.0 phage library (DNA-barcoded phages expressing overlapping 56mer peptides of more than 1500 different pathogens including 2263 peptides from six different EBV strains (AG876, B95-8, Cao, GD-1, P3HR-1, and Raji)) and IgG bound phages were immuno-precipitated using magnetic Protein A/G beads (Thermo Scientific Pierce). Phages were then lysed and DNA barcodes sequenced on an Illumina NextSeq 500 platform. NGS reads were aligned and respective peptides assigned to NGS counts. These peptides counts were then binned by the sum of four included negative (no plasma) controls. Epitope binding signal Z-scores for each sample and peptide were calculated per bin. For the Z-score calculation only the middle 90% (excluded the top and bottom 5%) of the values per bin were used. Peptides were only called ‘hit’ and further considered if they had an epitope binding signal Z-score of more than 3.5 in both replicates. For peptides with a hit at one timepoint this threshold was lowered to >2 for the other time point for the same peptide.

### Statistics

Flow cytometry data was quantified using FlowJo (FlowJo, LLC). Statistical analysis and graphs were generated using Prism (GraphPad). Radar plots were generated with the average of the Z-scored data per variable and group using the fsmb package (v.0.7) in R (v.3.6.1) and R Studio (v.1.3). For large data sets the reproducibility of results was confirmed by calculation of Pearson correlation coefficients. For pairwise comparisons, a two-tailed Mann-Whitney test or two-tailed paired T-test was applied. For multiple comparisons, a one-way ANOVA test with Tukey’s correction for multiple comparisons was used. The p values are depicted by * = p < 0.05, ** = p < 0.01, *** = p < 0.001, **** = p < 0.0001. Colors representing results for individual cohort participants were kept consistently throughout all figures of the article.

## Supporting information

S1 Fig Antibody responses to EBV of cohort participants measured by standard diagnostic ELISA. Detailed depiction of the data shown in Fig 1A on a per individual level. Different colors identify specific individuals. Every dot represents the signal measured for that time point and grey bars indicate the median signal.
(PDF)

S2 Fig Influenza HA1-specific IgM and IgG mediated ADCD and ADNP function. Detailed depiction of the data shown in Fig 2A on a per individual level **(A)** and Fig 2B (IgG1: **(B)**, IgG3: **(C)**). Grey dots refer to the average signal of reference samples of chronically EBV infected individuals (C-EBV+) or EBV uninfected controls (EBV-). Otherwise colored dots refer to the average signal of specific cohort individuals and time points of sample collection. Horizontal black lines show the median signals.
(PDF)

S3 Fig Influenza HA1-specific IgM and IgG mediated ADCD and ADNP function. The ability of influenza HA1-specific IgM and IgG antibodies samples from early (<4 wpe) and late (>24wpe) timepoints to mediate ADCD **(A)** and ADNP **(B)** was determined. Assays were performed with naïve (unmodified) serum of with serum that had been depleted of HA1-specific IgM or IgG as indicated. Every dot represents the average measured signal of a given sample. Significant differences in the levels of activity before and after depletion of IgM or IgG were determined by two-tailed T-test.
(PDF)

S4 Fig Respiratory syncytial virus (RSV)-, influenza- and rhinovirus-specific Ab responses in comparsion to EBV-specific Ab responses as determined by VirScan. Demonstrated are viral epitope binding signal Z-scores as a heatmap for an early timepoint (<4 wpe) and a late timpoint (>24 wpe) during EBV infection.
(PDF)

S5 Fig High SOI is associated with increased levels of EBV-specific IgM and Fc-functionality of p18-specific Abs. **(A)** For all four EBV antigens, IgM titer data was separated based on the either low (0–1) or high (2–6) self-reported SOI score of the corresponding individual at the same time point. Dots represent individual data points and black lines the median. The potential relationship between SOI and antigen-specific IgM titer was calculated using a two-tailed Mann-Whitney test. **(B)** The data describing ADNP and ADCD mediated by p18-specific antibodies was categorized and statistically analyzed as described for (A).
(PDF)
