# Supplementary figures and images for: Evolution of functional antibodies following acute Epstein-Barr virus infection
Source: PLoS Pathog. 2022 Sep 6;18(9):e1010738. doi: 10.1371/journal.ppat.1010738 (PMC9481173; doi:10.1371/journal.ppat.1010738)

**VCA IgM**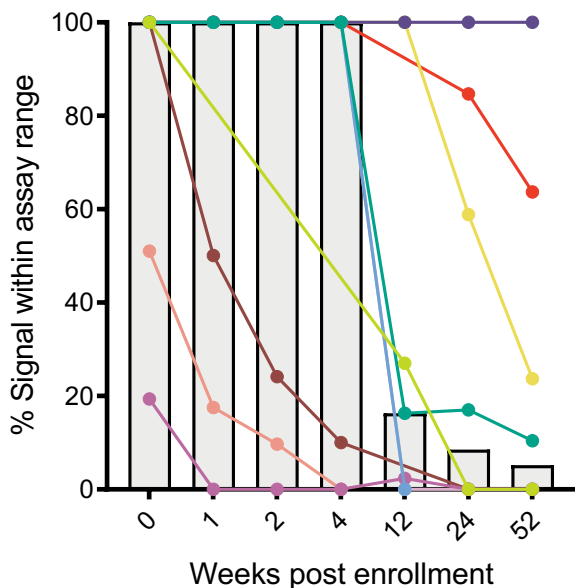**VCA IgG**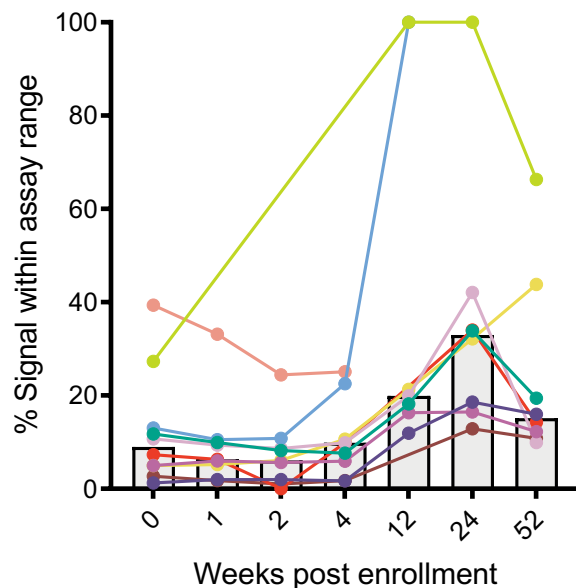

Supplement: S1 Fig — Detailed depiction of the data shown in Fig 1A on a per individual level. Different colors identify specific individuals. Every dot represents the signal measured for that time point and grey bars indicate the median signal. (PDF) [file ppat.1010738.s001.pdf]

**A**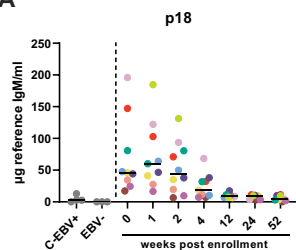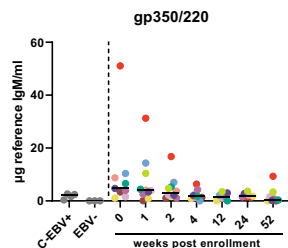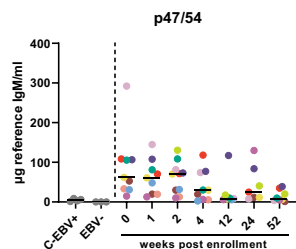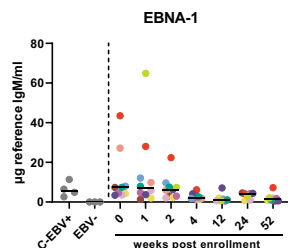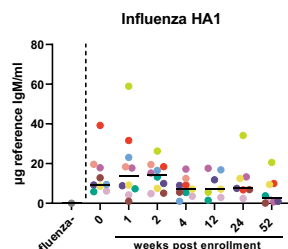**B**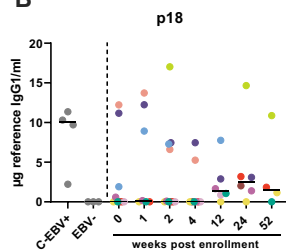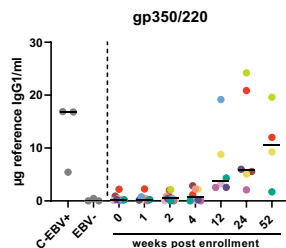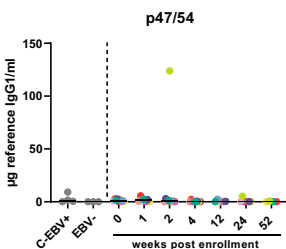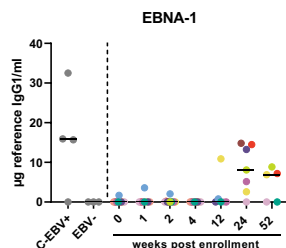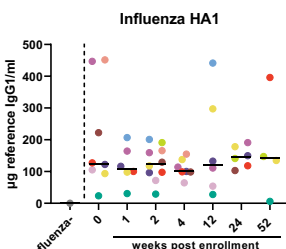**C**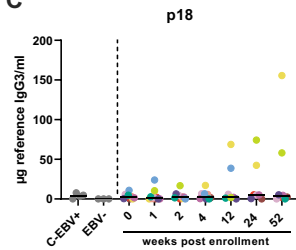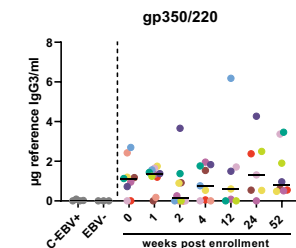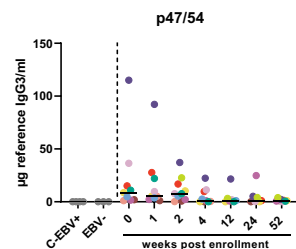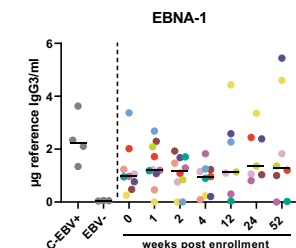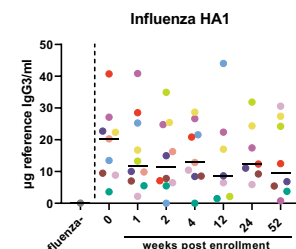

Supplement: S2 Fig — Detailed depiction of the data shown in Fig 2A on a per individual level (A) and Fig 2B (IgG1: (B), IgG3: (C)). Grey dots refer to the average signal of reference samples of chronically EBV infected individuals (C-EBV+) or EBV uninfected controls (EBV-). Otherwise colored dots refer to the average signal of specific cohort individuals and time points of sample collection. Horizontal black lines show the median signals. (PDF) [file ppat.1010738.s002.pdf]

**A**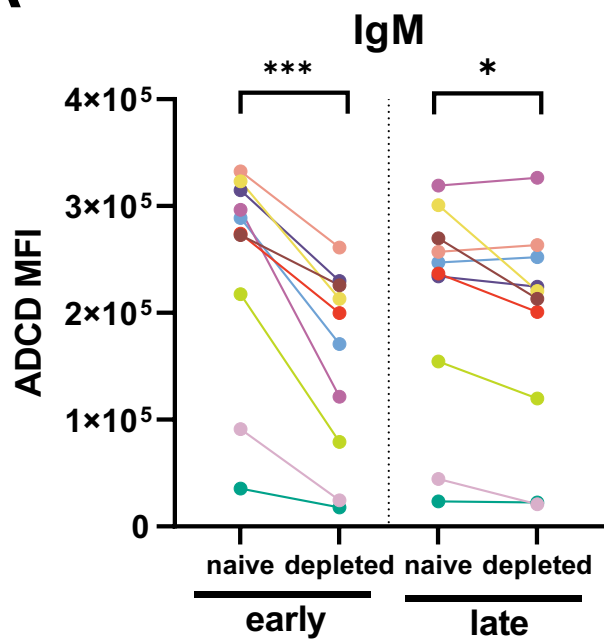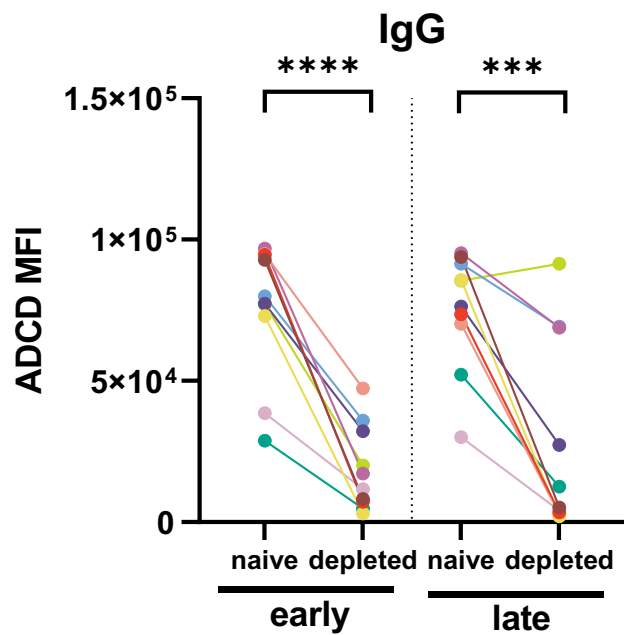**B**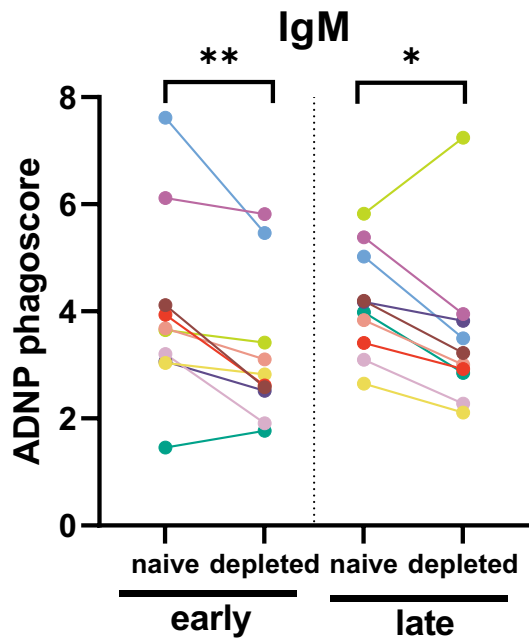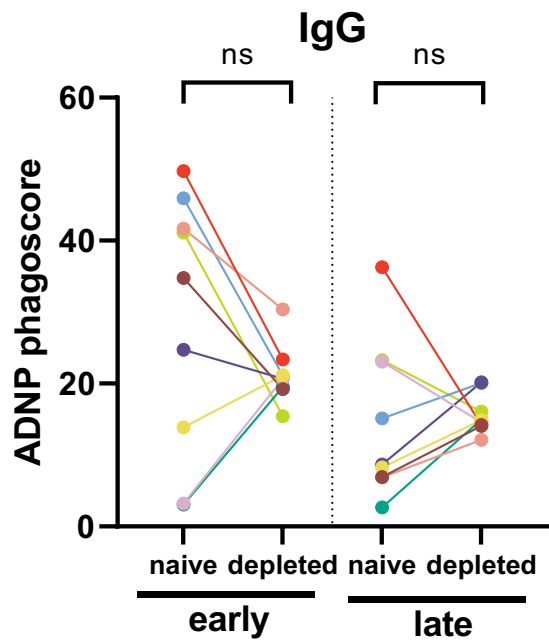

Supplement: S3 Fig — The ability of influenza HA1-specific IgM and IgG antibodies samples from early (<4 wpe) and late (>24wpe) timepoints to mediate ADCD (A) and ADNP (B) was determined. Assays were performed with naïve (unmodified) serum of with serum that had been depleted of HA1-specific IgM or IgG as indicated. Every dot represents the average measured signal of a given sample. Significant differences in the levels of activity before and after depletion of IgM or IgG were determined by two-tailed T-test. (PDF) [file ppat.1010738.s003.pdf]

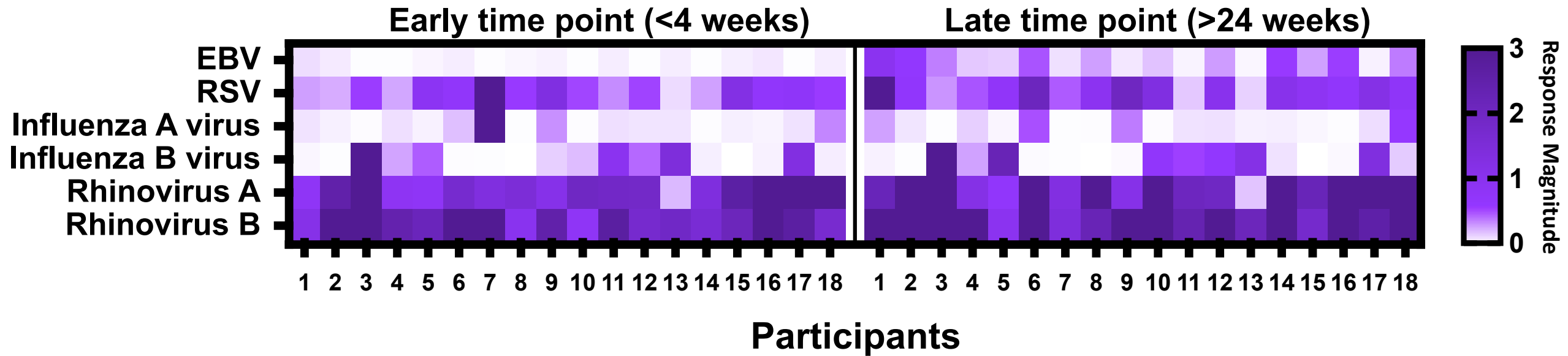

Supplement: S4 Fig — Demonstrated are viral epitope binding signal Z-scores as a heatmap for an early timepoint (<4 wpe) and a late timpoint (>24 wpe) during EBV infection. (PDF) [file ppat.1010738.s004.pdf]

**A**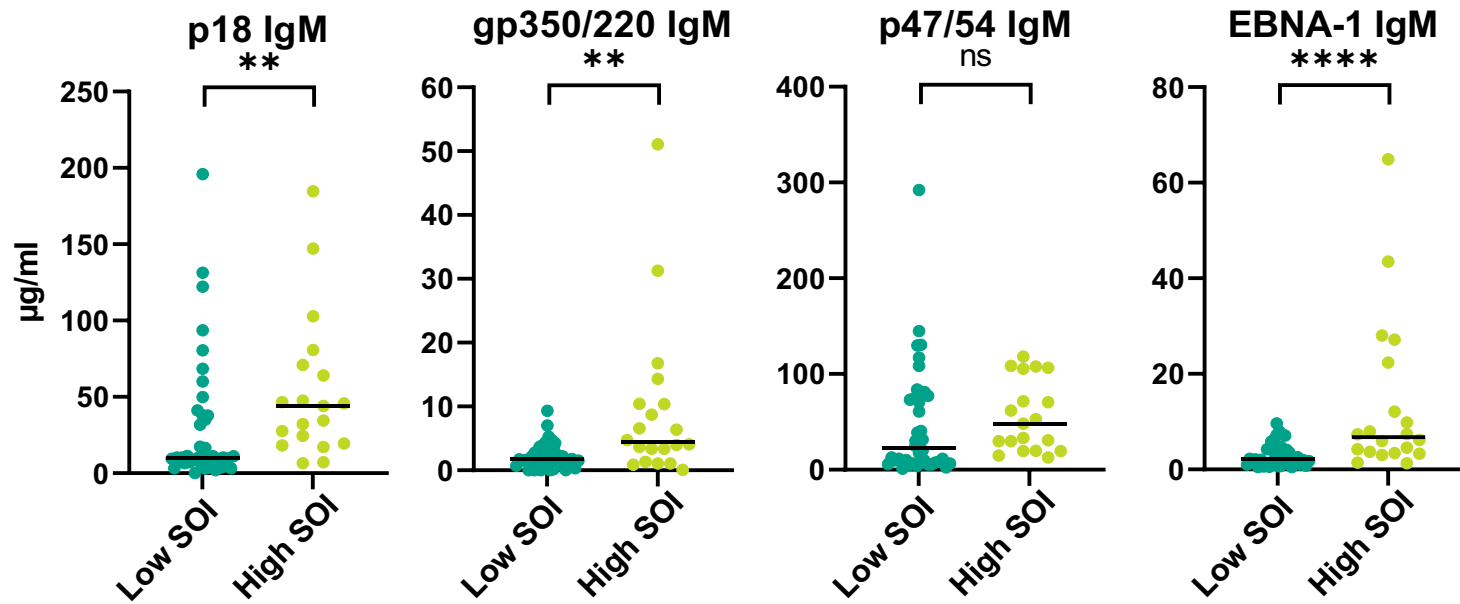**B**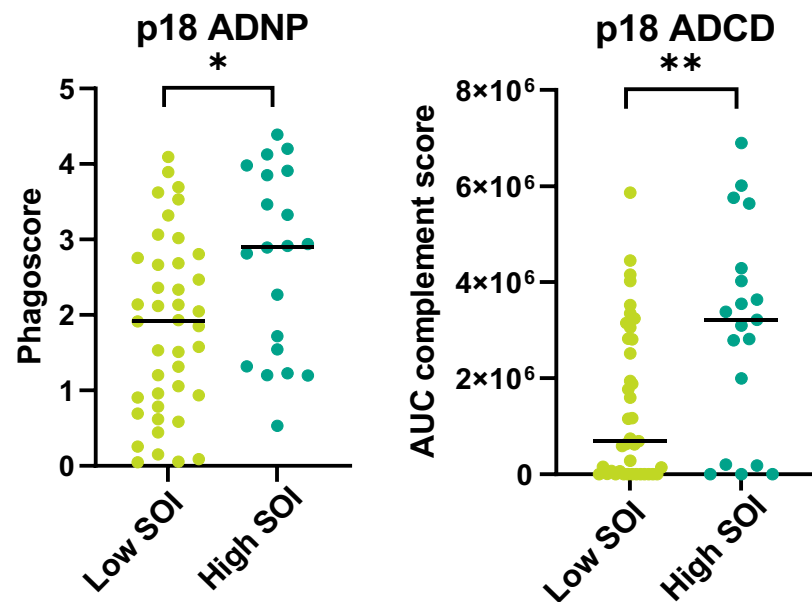

Supplement: S5 Fig — (A) For all four EBV antigens, IgM titer data was separated based on the either low (0–1) or high (2–6) self-reported SOI score of the corresponding individual at the same time point. Dots represent individual data points and black lines the median. The potential relationship between SOI and antigen-specific IgM titer was calculated using a two-tailed Mann-Whitney test. (B) The data describing ADNP and ADCD mediated by p18-specific antibodies was categorized and statistically analyzed as described for (A). (PDF) [file ppat.1010738.s005.pdf]
